# Portuguese propolis disturbs glycolytic metabolism of human colorectal cancer *in vitro*

**DOI:** 10.1186/1472-6882-13-184

**Published:** 2013-07-19

**Authors:** Isabel Valença, Filipa Morais-Santos, Vera Miranda-Gonçalves, Ana Margarida Ferreira, Cristina Almeida-Aguiar, Fátima Baltazar

**Affiliations:** 1 Life and Health Sciences Research Institute (ICVS), School of Health Sciences, University of Minho, Braga, Portugal; 2 ICVS/3B’s - PT Government Associate Laboratory, Braga/Guimarães, Portugal; 3 CBMA - Centre of Molecular and Environmental Biology, Biology Department, University of Minho, Braga, Portugal; 4 Chemistry Centre Vila Real (CQVR), University of Trás-os-Montes e Alto Douro, Vila Real, Portugal

**Keywords:** Antitumor activity, Colorectal cancer, Glycolytic metabolism, HCT-15 cells, Propolis

## Abstract

**Background:**

Propolis is a resin collected by bees from plant buds and exudates, which is further processed through the activity of bee enzymes. Propolis has been shown to possess many biological and pharmacological properties, such as antimicrobial, antioxidant, immunostimulant and antitumor activities. Due to this bioactivity profile, this resin can become an alternative, economic and safe source of natural bioactive compounds.

Antitumor action has been reported *in vitro* and *in vivo* for propolis extracts or its isolated compounds; however, Portuguese propolis has been little explored. The aim of this work was to evaluate the *in vitro* antitumor activity of Portuguese propolis on the human colon carcinoma cell line HCT-15, assessing the effect of different fractions (hexane, chloroform and ethanol residual) of a propolis ethanol extract on cell viability, proliferation, metabolism and death.

**Methods:**

Propolis from Angra do Heroísmo (Azores) was extracted with ethanol and sequentially fractionated in solvents with increasing polarity, n-hexane and chloroform. To assess cell viability, cell proliferation and cell death, Sulforhodamine B, BrDU incorporation assay and Anexin V/Propidium iodide were used, respectively. Glycolytic metabolism was estimated using specific kits.

**Results:**

All propolis samples exhibited a cytotoxic effect against tumor cells, in a dose- and time-dependent way. Chloroform fraction, the most enriched in phenolic compounds, appears to be the most active, both in terms of inhibition of viability and cell death. Data also show that this cytotoxicity involves disturbance in tumor cell glycolytic metabolism, seen by a decrease in glucose consumption and lactate production.

**Conclusion:**

Our results show that Portuguese propolis from Angra do Heroísmo (Azores) can be a potential therapeutic agent against human colorectal cancer.

## Background

Propolis is a resin containing a complex mixture of compounds that honeybees collect from several plants, further digest with salivary enzymes and mix with beeswax. Propolis is composed mainly by wax and resin, but also contains essential oils, pollen, phenolic acids or their esters, flavonoids, terpenes, aromatic aldehydes, alcohols, and fatty acids, among others [1]. Propolis composition depends on various factors such as the source of plant species from which propolis is made and on environmental factors, which constitute a difficulty to its standardization [2,3]. For instance, Brazilian propolis is composed essentially by prenylated *p*-coumaric acids and diterpenic acids [4], whereas propolis of temperate zones is mainly composed by flavones, flavanones, cinnamic acids and their esters [2,5]. Although scarcely studied, Portuguese propolis appears to be composed by the typical phenolic acids and flavonoids found in samples of temperate zones, especially in Europe, but it contains also several other new compounds that had never been referred before [6].

Propolis has been extensively employed in folk medicine since ancient times. Recently, a wide range of biological and pharmacological actions have been demonstrated for several types of propolis, such as antibacterial [7,8], anti-fungal [8,9], anti-viral [10], anti-inflammatory [11,12], antioxidant [13,14], hepatoprotective [15], immunostimulant [16] and antitumor activities [13,17-19], among others.

Portuguese propolis pharmacological properties have been little explored. In fact, this resin has been highly neglected by both beekeepers and the scientific community. Only its chemical composition [6,20], antioxidant activity [13,20,21] and antitumor activity on renal cell carcinoma [13] have been studied on a few samples of some regions of Portugal. These few studies revealed diversity in terms of chemical composition and biological properties, as well as the presence of some compounds never found in propolis from other origins [6], renewing the interest in studying this natural compound.

Thus, the aim of this work was to assess and characterize the antitumor activity of a propolis sample collected in Angra do Heroísmo (Archipelago of Azores, Portugal) in a colon cancer cell line, including its effects on tumour cell glycolytic metabolism. Despite evidence that some compounds occasionally present in propolis are able to inhibit lactate transport [22-26], to the best of our knowledge, propolis potential in disturbing cancer cell metabolism was never investigated.

## Methods

### Preparation of propolis extracts and fractions

Propolis was collected in March 2009 from *Apis mellifera* beehives located in Angra do Heroísmo (AH), Azores, Portugal. Propolis (40 g) was frozen at −18°C, grounded and extracted at room temperature with ethanol under slow stirring. The obtained solution was filtered and re-extracted twice more under the same conditions, giving the ethanol extract (EE) after filtration and solvent evaporation. This sample was designated AH.EE.09. The extract was further and sequentially fractionated in solvents with increasing polarity - n-hexane (H) and chloroform (C) – yielding, after solvent removal at 40°C under vacuum, the hexane (AH.FH.09) and the chloroform fractions (AH.FC.09) of propolis and a residual ethanol extract fraction (AH.FEr.09). Dried fractions were stored at 4°C and were diluted in DMSO to obtain the working solutions at the desired concentrations.

### Determination of total polyphenol and flavonoid contents

Total phenolic content was determined according to the Folin–Ciocalteu colorimetric method [27], with some modifications, and using gallic acid as standard.

Total flavonoid content was quantified according to the method described by Woisky and Salatino [28], with some modifications. Quercetin was used as standard for total flavonoid content quantification.

### Cell culture

The experiments were performed on the human colorectal adenocarcinoma cell line HCT-15. Cells were grown in RPMI 1640 medium (Gibco, Invitrogen, USA) supplemented with 10% fetal bovine serum (Gibco, Invitrogen, USA) and 1% penicillin/streptomycin (Invitrogen, USA). Cells were incubated at 37°C in atmosphere containing 5% CO_2_.

### Cell viability assay

Cell viability was measured by the sulforhodamine B assay (Sigma Chemical Company, MO, USA) following the manufacturer’s instructions. HCT-15 cells were incubated in 96-well plates (1×10^4^ cells/well) with different concentrations (0.005 mg/ml – 0.05 mg/ml) of propolis samples for 24, 48 and 72 hours. Controls were treated with DMSO alone (1%).

Results are presented as mean ± SD of three independent experiments, each in triplicate. IC50 values were calculated for each propolis fraction and time point.

### Cell proliferation assay

Cell proliferation was measured using the 5-bromo-2′-deoxyuridine (BrdU) Cell Proliferation Assay (Roche, Mannheim, Germany). HCT-15 cells were incubated in 96-well plates (1×10^4^ cells/well) for 24 hours with the half maximal inhibitory concentrations (IC_50_s) and two higher concentrations (0.025 and 0.05 mg/ml) of AH.FC.09 and AH.FEr.09. Controls were treated with DMSO alone (1%). BrdU was added after 24 hours of propolis exposure and was quantified according to the manufacturer’s instructions using a microplate reader (Model 680, Bio Rad) at 450 nm.

Results are presented as mean ± SD of three independent experiments, each in triplicate.

### Cell metabolism assay

Extracellular glucose and lactate were measured using commercial kits for glucose (Cobas, Roche) and lactate (Spinreact, S.A.U.) according to the manufacturer’s protocols, but scaled down to microplate volumes. HCT-15 cells were incubated for 24 hours in 24-well plates (4×10^5^ cells/well) with the IC_50_ concentrations for AH.FC.09 and AH.FEr.09, at 24 hours, and two higher concentrations (see above). Controls were treated with DMSO alone (1%).

Results are presented as mean ± SD of three independent experiments, each in triplicate.

### Cell death assay

Apoptotic and necrotic cell populations were determined by Annexin V (BD Biosciences). Briefly, HCT-15 cells were seeded in T25 flasks and incubated until 90% confluence. Cells were treated with AH.FC.09 and AH.FEr.09 at IC_50_ concentrations for 24 hours. Controls were treated with DMSO alone (1%). After incubation, culture supernatant was recovered from each flask and treated cells were trypsinized. Cell pellets were ressuspended in 1 ml binding buffer (10 mM Hepes pH 7.4, 140 mM NaCl and 2.5 mM CaCl_2_) and centrifuged at 2000 rpm × 5 min. Then, cells were incubated 15 min. with staining solution (8 μl Annexin and 30 μl PI (50 μg/ml) per 100 μl of binding buffer) at room temperature. The percentage of cell death was assessed by flow cytometry (LSRII model, BD Biosciences): a total of 20,000 events was recovered and the results were analyzed using the FlowJo software (version 7.6; Tree Star, Inc.).

Results are presented as mean ± SD of three independent experiments.

### Statistical analysis

Statistic analysis was performed using the GraphPad Prism. Unpaired *t*-test was performed to compare two groups. Significance was considered as *p* < 0.05.

## Results

### Propolis polyphenol and flavonoid contents

The values for total polyphenol and flavonoid contents (Table 1) show that AH.FC.09 has the greatest amount (214.8 mg/ml) whereas AH.FH.09 exhibits the lowest amount (134.3 mg/ml of polyphenols). Concerning flavonoid content, AH.FC.09 and AH.FEr.09 have similar amounts - 25.5 and 26.9 mg/ml respectively - and AH.FH.09 presents the lowest amount of flavonoids (13.5 mg/ml).

**Table 1:** Total polyphenol and flavonoid contents of propolis were determined in all the fractions of Angra do Heroísmo propolis

	**Total polyphenols (mg/ml)**	**Total flavonoids (mg/ml)**
**AH. FH.09**^**a)**^	134.3 ± 1.3	13.5 ± 0.4
**AH.FC.09**^**b)**^	214.8 ± 9.1	25.5 ± 0.6
**AH.FEr.09**^**c)**^	194.0 ± 4.7	26.9 ± 0.4

Each value indicates mean ± SD.

a) Hexane fraction.

b) Chloroform fraction.

c) Ethanol residual fraction.

### Effect of propolis fractions on cell viability

**Figure 1:**
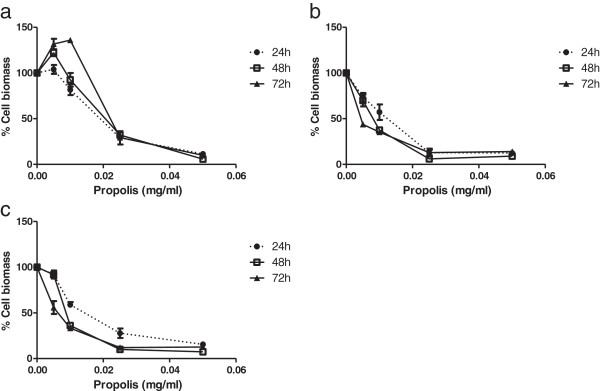
**Effect of propolis on HCT-15 cell viability.** HCT-15 cells were treated for 24, 48 and 72 h with different concentrations of propolis fractions - AH.FH.09 **(a)**, AH.FC.09 **(b)** and AH.FEr.09 **(c)** - and control cells were treated with DMSO alone. Results are expressed as percentage of cell biomass.

The effect of propolis fractions on the viability of HCT-15 cells was assessed by the sulforhodamine B assay (Figure 1). Cells were incubated for 24, 48 and 72 hours with different concentrations of propolis fractions. All propolis samples led to a decrease in cell biomass of HCT-15 cells in a dose- and time-dependent manner, with IC_50_ values ranging from 0.005 to 0.026 mg/ml (Figure 1 and Table 2). AH.FC.09 appears to be the most effective and AH.FH.09 the least effective in decreasing cell viability (Figure 1). At low concentrations AH.FH.09 seems to increase cell biomass, mainly after 48 and 72 hours incubation (Figure 1a).

**Table 2:** **IC**_**50 **_**values of propolis fractions - AH.FH.09, AH.FC.09 and AH.FEr.09 - on HCT-15 cell line**

	**AH.FH.09**^**a)**^**(mg/ml)**	**AH.FC.09**^**b)**^**(mg/ml)**	**AH.FEr.09**^**c)**^**(mg/ml)**
**24 h**	0.020	0.01	0.015
**48 h**	0.024	0.007	0.009
**72 h**	0.026	0.005	0.005

a) Hexane fraction of Angra do Heroísmo propolis collected in 2009.

b) Chloroform fraction of Angra do Heroísmo propolis collected in 2009.

c) Ethanol residual fraction of Angra do Heroísmo propolis collected in 2009.

### Effect of propolis fractions on cell proliferation

To assess the effect of propolis on cell proliferation, HCT-15 cells were treated with IC_50_ concentrations, as well as two higher concentrations of the most effective fractions, AH.FC.09 and AH.FEr.09.

**Figure 2:**
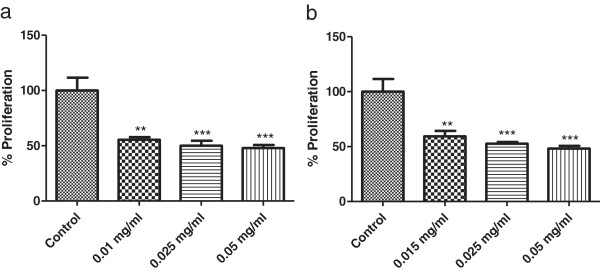
**Effect of different concentrations of propolis fractions - AH.FC.09 (a) and AH.FEr.09 (b) - on HCT-15 cell proliferation.** Controls were treated with DMSO alone. Results are presented as percentage of proliferation. ** p < 0.01 vs control. *** p < 0.001 vs control.

Both fractions were able to decrease HCT-15 cell proliferation (Figure 2). All tested concentrations of propolis led to a decrease of approximately 40-60% in cell proliferation after 24 hours of incubation.

### Effect of propolis fractions on cell metabolism

**Figure 3:**
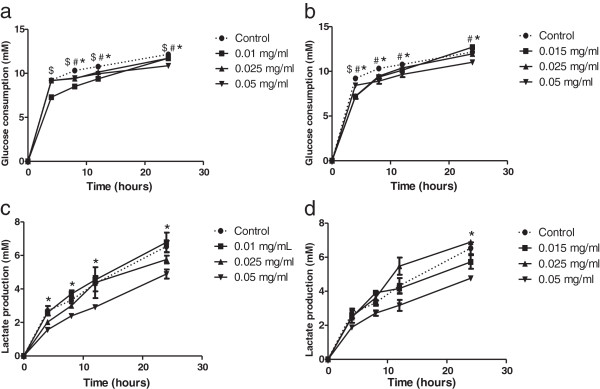
**Effect of propolis on HCT-15 cell metabolism.** HCT-15 cells were treated with several concentrations of AH.FC.09 **(a,c)** and AH.FEr.09 **(b,d)**. Controls were treated with DMSO alone. Results are presented as glucose consumption **(a,b)** and lactate production **(c,d)**. ^$^ p < 0.05, 0.01/0.015 mg/ml vs control; ^#^ p < 0.05, 0.025 mg/ml vs control; ^*^ p < 0.05 0.05 mg/ml vs control.

After 24 hours of incubation with different concentrations (IC_50_ and two higher concentrations) of AH.FC.09 and AH.FEr.09, there was a decrease in the glycolytic metabolism of HCT-15 cells (Figure 3). AH.FC.09 led to a decrease in glucose consumption and lactate production along 24 hours of incubation (Figure 3a,c). AH.FEr.09 led to a decrease in glucose consumption along 24 hours, however, lactate production only decreased after 24 hours of incubation with 0.05 mg/ml (Figure 3b,d).

### Effect of propolis fractions on cell death

**Figure 4:**
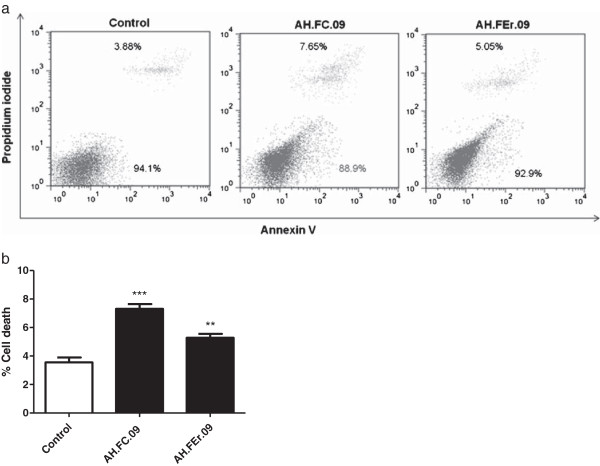
**Effect of propolis on HCT-15 cell death.** Representative dotplot of cell population distribution stained for Annexin V and PI **(a)** and graphic representation **(b)**. Cells were treated with IC_50_ concentrations for 24 hours and control was treated with DMSO alone. Results are presented as percentage of cell death. ** p < 0.01 vs control. *** p < 0.001 vs control.

To evaluate the effect of propolis on cell death, HCT-15 cells were incubated with the IC_50_ values of each fraction for 24 hours. AH.FC.09 and AH.FEr.09 fractions moderately induced cell death on HCT-15 cells, about 7% and 5%, respectively (Figure 4).

## Discussion

Propolis has been widely studied for its biological properties and has been used by several industries, such as food, cosmetics and pharmaceutical industries [2,29]. *In vitro* and *in vivo* studies on propolis antitumor activity have been widely reported [13,18,30-35], however, there is only one study exploring Portuguese propolis with this purpose [13,18,30-35], where the authors reported inhibition of human renal cancer cell growth.

In the present study, we aimed to assess the antitumor activity of a fractionated Portuguese propolis sample collected in Angra do Heroísmo, an island of the Azores Archipelago, on HCT-15 colon cancer cell line. This is the first study to assess antitumor activity in this tumor model and with Portuguese propolis.

Our results showed that all propolis fractions were able to decrease cell viability of colon tumor cells (Figure 1), being in accordance with previous reports [30,32-35]. However, lower concentrations of the hexane fraction seem to increase the number of viable cells (Figure 1a). Propolis is a mixture of several compounds from plants and since this solvent has low polarity, the hexane fraction has lower amounts of dissolved polyphenols and flavonoids than chloroform or ethanol fractions (Table 1), both considered the main bioactive compounds in European propolis. This is probably why this fraction was not able to decrease cell viability when tested at low concentration. The chloroform fraction seems to be more powerful in decreasing cell viability (Figure 1b), which may be due to the presence of superior amounts of compounds of higher polarity, like the phenolic compounds (Table 1), which are more efficiently extracted with chloroform than hexane.

Since AH.FC.09 and AH.FEr.09 were the most effective fractions, we further assessed its effects on cell proliferation, metabolism and death. The results show that propolis led to a decrease in HCT-15 cell proliferation (Figure 2), as described in other studies [33-35]. Regarding the effect of propolis on cell death, there was a small induction in cell death (Figure 4), again in accordance with results of other reports concerning other propolis samples [30,31]. Ishihara *et al.*[30] showed that Chinese and Brazilian propolis induced apoptosis in cell cultures of human colon carcinoma cells. Also, Szliszka *et al.*[31] showed that tumor necrosis factor-related apoptosis-inducing ligand (TRAIL) in prostate cancer cells is markedly augmented by a propolis ethanol extract from Poland.

The cytotoxic effect of the studied propolis fractions may be related to the overall effect of their phenolic compounds. Over the last decades, several phenolic compounds (mainly flavonoids and phenolic acids) have been linked to propolis antitumor activity against colorectal tumor cells *in vitro*. Among flavonoids, quercetin is able to inhibit cell growth with cytotoxic activity [36,37], to reduce cell proliferation [38], induce cell cycle arrest and apoptosis [37,39]. Apigenin, another flavonoid, can induce cell cycle arrest and apoptosis [40,41]; rutin is able to inhibit cell proliferation and induce apoptosis [42,43]; galangin shows antiproliferative capacities [35] and chrysin is able to inhibit cell growth, reduce cell proliferation, induce cell cycle arrest and apoptosis [35,42,43]. In what concerns phenolic acids, cinnamic acid displayed antiproliferative effects [35,44] and caffeic acid phenethyl ester (CAPE) inhibited proliferation [35] and induced growth arrest and apoptosis [45].

To the best of our knowledge, propolis effect on glycolytic metabolism of cancer cells has not yet been investigated. The present study shows for the first time that a Portuguese propolis sample has antitumor activity on HCT-15 colon cancer cell line and that such activity likely involves disturbance of tumor cell metabolism, as assessed by the effects on the rates of glucose consumption and lactate production (Figure 3). It is known that cancer cells have preference for glycolytic metabolism, displaying higher glycolytic rates than those of normal cells [46]. In normal cells and in the presence of oxygen, pyruvate undergoes oxidative phosphorylation, leading to highly efficient energy production in the form of ATP. However, if oxygen levels are low, pyruvate is converted into lactate in the cytoplasm, a process much less efficient in terms of energy production. In the case of tumor cells, pyruvate is preferentially converted to lactate even in the presence of oxygen, a process known as the Warburg effect [46]. To maintain intracellular pH, and to avoid cell death, tumor cells use beyond other pH regulators, monocarboxylate transporters (MCTs) for proton-coupled lactate efflux and maintenance of glycolytic rates [47]. Therefore, if the activity of these transporters is inhibited, there will be an increase of intracellular lactate and consequent impairment of glycolysis, with a consequent decrease in glucose consumption [48].

Although propolis effect on glycolytic metabolism of cancer cells has not yet been investigated, there are some studies that associate a wide range of phenolic compounds, some present in propolis samples, to glycolytic metabolism inhibition. Shim *et al*. [22] showed that the flavonoids naringenin, morin, silybin and quercetin are competitive inhibitors of MCT1 in Caco-2 colon cancer cells. Also, Wang *et al.*[23] showed that several flavonoids, such as apigenin, biochanin A, chrysin, diosemin, fisetin, genistein, hesperitin, kaempferol, luteolin, morin, narigenin, phloretin, and quercetin may significantly alter the pharmacokinetics and pharmacodynamics of MCT1 substrates. Phenolic acids can also be responsible for MCT inhibition. *Alpha*-cyano-4-hydroxycinnamic acid, a cinnamic acid derivative, has been associated with MCT activity inhibition in several tumor models [24-26]. However, further studies are needed to understand the mechanism by which propolis affects cell glycolytic metabolism, namely if it is mediated by inhibition of lactate transport through MCTs.

## Conclusions

The cytotoxicity of the Portuguese propolis sample studied appears to be due not only to a decrease in cell proliferation and induction of cell death but also to disturbance of cancer cell glycolytic metabolism. Overall, these results support that Portuguese propolis or its components should be further explored as therapeutic agents in the treatment of cancer.

## Competing interests

The authors declare no conflicts of interest.

## Authors’ contributions

IV conducted most of the experimental work and wrote the first draft of the manuscript. FMS supervised and helped in the in vitro assays. VG performed the cytometry studies and helped writing the manuscript. AMF provided and prepared propolis samples. CAA and FB conceived and designed the study, coordinated its development and corrected the manuscript. All authors read and approved the final manuscript.

## Pre-publication history

The pre-publication history for this paper can be accessed here:

http://www.biomedcentral.com/1472-6882/13/184/prepub
